# Supplementary material for: Road extraction through Yangwang-1 nighttime light data: A case study in Wenzhou, China
Source: PLoS One. 2024 Jan 19;19(1):e0297152. doi: 10.1371/journal.pone.0297152 (PMC10798492; doi:10.1371/journal.pone.0297152)
Supplement: Attachment — Submitted filename: Comments_by-Seyoum Eshetie.pdf [file pone.0297152.s001.pdf]

<u>Comments for Manuscript PONE-D-23-26958</u>

1. The Title is doesn't align with the objectives and findings of the research.
2. The novelty of this paper is not clear. The difference between present work and previous Works should be highlighted. Also, needs to include similar previous studies and discuss them.

   The following paragraphs do not show research gap.

   Line [106-108]

   > *This study aims to develop an improved U-Net model (CA U-Net) combined with Cross-Attention Mechanisms, tailored for road extraction from remote sensing images by incorporating modifications that enhance its performance in identifying and delineating road networks. We hypothesize that the unique properties of NTL data, combined with the powerful feature learning capabilities of deep learning models, can lead to accurate and effective road extraction.*

   Line [177-180]

   > *The U-Net framework, paired with CAM, provides an advanced methodology for road extraction from nighttime light (NTL) data. This deep learning architecture has been extensively used in remote sensing image semantic segmentation and classification tasks, due to its proficiency in capturing both global and local features with its symmetric encoder-decoder structure.*
3. Section 3.1 should be relocated to Section 2
4. Data preparation and preprocessing is not well explained.
5. Generalized research method workflow is missing
6. Software and/programs used in the research are not included
7. The paper lacks a thorough comparison of the cited works and a detailed discussion of the findings.
8. CONCLUSIONS is more of an afterthought. The authors are suggested to include afterthought of this work.
9. Recommend some avenues for future research at the end of the conclusion section
