# Supplementary material for: Road extraction through Yangwang-1 nighttime light data: A case study in Wenzhou, China
Source: PLoS One. 2024 Jan 19;19(1):e0297152. doi: 10.1371/journal.pone.0297152 (PMC10798492; doi:10.1371/journal.pone.0297152)
Supplement: Attachment — Submitted filename: Response to Reviewer 2.docx [file pone.0297152.s002.docx]

Response to the Reviewer 2 on PONE-D-23-26958

Title: “**Road Extraction Through Yangwang-1 Nighttime Light Data: A Case Study in Wenzhou, China**”

Dear Reviewer,

I would like to express my gratitude to you and the reviewers for taking the time to thoroughly review my manuscript “Road Extraction Through Yangwang-1 Nighttime Light Data: A Case Study in Wenzhou, China”. I appreciate the valuable feedback provided, and I have addressed all the comments and suggestions to improve the quality of the manuscript. Please find below a summary of the major revisions made in response to your comments. And the revised part in our manuscript has been highlighted with **red** color.

1. *The Title is doesn’t align with the objectives and findings of the research*

**Our response:** Thanks for your helpful comments, we have revised the title:

Road Extraction Through Yangwang-1 Nighttime Light Data: A Case Study in Wenzhou, China

2. The novelty of this paper is not clear. The difference between present work and previous works should be highlighted. Also, needs to include similar previous studies and discuss them. The following paragraphs do not show research gap Line [106-108] and Line [177-180].

**Our response:** Thanks for your helpful suggestions. We have made thoroughly revision *Introduction* to compare with the previous work, document the challenges of current research and conclude the research gaps. We also present the contributions and innovations of our study. Some evidence is as follows:

**Introduction (Line 46-56):** Current approaches predominantly depend on the expensive high-resolution satellite images and are also struggled with the complex nature of urban and rural environments, which involve varying road materials, occlusions by buildings and vegetation, shadow effects, and diverse terrain conditions. These ground features often occupy more than 80% pixels of one satellite images and their texture and spectral features will significantly cause impacts on the quality and accuracy of the extracted roads. These challenges often stem from the complexity and diversity of road features, as well as the varying quality and resolution of satellite imagery. Other environmental factors, such as shadows of ground objects, can also impact the effectiveness of road extraction algorithms[8]. Therefore, we believe it's crucial to utilize a new type of data sources rich in road information, while also being cost-effective in the extraction of road information.

**Introduction (Line 80-113):** Despite the numerous successful applications of Night-Time Light (NTL) data in socioeconomic studies, its use in road extraction remains relatively unexplored. Only a handful of studies have endeavored to employ NTL images for road extraction tasks, achieving some noteworthy results. For instance, Wang [18] harnessed Luojia 1-01 NTL imagery to extract roads in Wuhan through shape optimization and a thresholding approach, attaining a final extraction accuracy of 83.2%. However, the resolution of Luojia 1-01 NTL data is relatively low. In contrast, the higher resolution (30 m) Sustainable Development Goals Satellite-1 (SDGAST) was utilized in Chang's [9] research, where a watershed segmentation approach was employed to extract roads in Beijing and Wuhan. This method yielded an extraction accuracy of 84.65% when compared to OpenStreetMap (OSM) road network data. Although these studies underscore the potential and feasibility of using NTL data for road extraction, they also highlight several inherent limitations and challenges. (1) The adoption of NTL data for ground object extraction tasks remains limited—a possible reason might be that the majority of NTL research focuses on the use of coarse-resolution NTL images such as VIIRS and DMSP data. This focus is largely due to their free accessibility and straightforward processing workflow. However, these coarse-resolution NTL datasets often fall short in detecting the edges of ground objects, such as roads, thereby posing significant challenges in road extraction tasks. The overglow effect - where light from urban areas spills over to adjacent regions - can result in the overestimation of road extents in these rough-resolution images[9]. Moreover, the relatively coarse spatial resolution of NTL data compared to high-resolution daytime imagery can limit the ability to capture detailed road features. That also makes the usage of NTL data mostly concentrated on socioeconomic aspects. Nowadays, more NTL satellites with higher resolution have been launched, benefitting from the technical advancement and the prospective application of NTL data in sustainable development goals. The emergency of these new NTL images gradually makes ground objection extraction possible with low cost. However, the study on these new generation NTL data is still lacking. (2) Some external data sources, like OSM data, taxi GPS data, and smartphone GPS data, are often combined with VHR imageries when performing road extraction [19-21], however, that rarely happens during the analysis of NTL data set. A very important reason is that the previous NTL data do not have the capacity to match with the external data sources due to low resolution. (3) The analysis method of current NTL data is also very simple – mainly threshold-based methods and morphology-based methods, which can be sufficient for macroscopical issues, like GDP estimation, population estimation and consumption potential prediction [22,23]. But these methods are not enough to solve the objective recognition tasks. Therefore, another challenge is to improve the current NTL analysis methods to adapt to the new possibilities of these new generation NTL images.

**Introduction (Line 134-151)**: In addressing these research gaps and amplifying the accuracy of road extraction via NTL data, this study attempts to prove the feasibility of employing the new generation NTL satel-lite—Yangwang-1—in road extraction endeavors. We derive an improved U-Net model (CA U-Net) combined with cross-attention mechanisms to extract information from NTL images and incorporating characteristics from OpenStreetMap (OSM). We hypothesize that the unique properties of NTL data, combined with the powerful feature learning capabilities of deep learning models, can lead to accurate and effective road extraction. We also compare the ex-traction results with traditional road extraction methods—Support Vector Machines (SVM) and Optimal Threshold (OT), which do not use OSM data in training process, and further find that the proposed method can archive better performance (accuracy) than traditional methods. The main contributions of our work can be concluded as bellow: (1) We tested the capacity of new generation NTL imagery—Yangwang-1, in road extraction tasks. To our knowledge, it’s the first attempt in adopting 30m resolution NTL imagery in ground object extraction. (2) We firstly combine NTL data and the external data set—OSM data in the training process through a deep learning framework and cross-attention mechanisms. Our approach largely improves the road extraction accuracy of traditional threshold methods, which use NTL data only and the street network data (OSM) is just used for accuracy assessment. Our research can provide references for high-accuracy and low-cost road extraction through fine-resolution NTL image-ry.

8. Xin, J.; Zhang, X.; Zhang, Z.; Fang, W. Road extraction of high-resolution remote sensing images derived from DenseUNet. *Remote Sensing* **2019**, *11*, 2499.

9.	Chang, D.; Wang, Q.; Yang, J.; Xu, W. Research on road extraction method based on sustainable development goals satellite-1 nighttime light data. *Remote Sensing* **2022**, *14*, 6015.

18. Wang, L.; Zhang, H.; Xu, H.; Zhu, A.; Fan, H.; Wang, Y.J.A.S. Extraction of City Roads Using Luojia 1-01 Nighttime Light Data. **2021**, *11*, 10113.

19.	Wu, S.; Du, C.; Chen, H.; Xu, Y.; Guo, N.; Jing, N.J.I.I.J.o.G.-I. Road extraction from very high resolution images using weakly labeled OpenStreetMap centerline. **2019**, *8*, 478.

20.	Li, Y.; Xiang, L.; Zhang, C.; Jiao, F.; Wu, C.J.I.J.o.S.T.i.A.E.O.; Sensing, R. A guided deep learning approach for joint road extraction and intersection detection from RS images and taxi trajectories. **2021**, *14*, 8008-8018.

21.	Du, R.; Qiu, G.; Gao, K.; Hu, L.; Liu, L.J.S. Abnormal road surface recognition based on smartphone acceleration sensor. **2020**, *20*, 451.

22.	Wang, L.; Fan, H.; Wang, Y.J.S.o.T.T.E. Improving population mapping using Luojia 1-01 nighttime light image and location-based social media data. **2020**, *730*, 139148.

23.	Wang, L.; Fan, H.; Wang, Y.J.P.o. Estimation of consumption potentiality using VIIRS night-time light data. **2018**, *13*, e0206230.

*3. Section 3.1 should be relocated to Section 2*

**Our response:** Thank you for your suggestions. We have moved the previous 3.1 section and 3.2 section to the current Section 2.1 *Study area* and Section 2.2 *Data sources and preprocessing*. Moreover, we have added more details of the processing steps and flowchart (Line 195-234).

4. *Data preparation and preprocessing is not well explained.*

**Our response:** Thank you for your helpful comments. We have made significant changes of this part and added more details about our data preparing and preprocessing. We also put a flowchart describing our data processing/road extraction steps:

**Section 2.2 (Line 195-234):** Following the acquisition of Yangwang-1 NTL and OSM data, a series of preparatory steps are necessitated prior to their deployment for road extraction. (1) Initially, both datasets are projected into the WGS-84 coordinate system, a pivotal step for preserving spatial integrity and ensuring alignment between them. (2) Subsequently, data registration is performed utilizing fifteen control points to ascertain the spatial congruity of these datasets. These control points are judiciously dispersed across the NTL imagery and predominantly selected from road junctions, given the paucity of other discernible ground objects within the NTL data. (3) Thereafter, pixels situated in water bodies and mountainous terrains are excised from the NTL data through spatial overlay with OSM land use data. (4) To mitigate adverse influences from background noise and the blooming effects inherent in NTL data, thresholds are established with a lower limit of 1 and an upper limit of 3325, the latter representing the maximal radiance value observed within the commercial center (pixels exhibiting 4095 radiance are identified as blooming effects). Consequently, pixels manifesting radiance exceeding 3325 are normalized to 3325. This thresholding strategy is devised to preserve all prospective road pixels whilst eliminating non-lit areas or noise, thus honing the focus on the urban roads of interest. (4) Subsequently, the original roads are transmuted into raster data congruent with the resolution of NTL data (30m resolution), with pixels being registered with NTL data. Within the raster data, pixels are bifurcated into road pixels and non-road pixels. (5) The Yangwang-1 NTL data alongside the road raster data are then partitioned into 1462 pairs of image segments, each encompassing $32\times32$ pixels. (6) Finally, these images are stochastically apportioned into training samples comprising 1022 pairs of images, and a test set constituting 440 pairs of images, thereby priming the data for ensuing analytical endeavors.

**Figure 2**. Flowchart of the road extraction process.

The flowchart of this study is shown in Figure 2, in which three main steps are included: (1) Data Preprocessing. The Yangwang-1 NTL imagery and OSM data are projected into a uniform WGS-84 coordinate system, and undesirable pixels within the NTL imagery are expunged. Subsequently, OSM roads are transmuted into raster data, delineating between road pixels and non-road pixels. The NTL data and OSM raster data are then bifurcated into paired segments, primed for data training. (2) Data Training and Road Extraction. The segmented smaller NTL images and corresponding road segments are stochastically selected to constitute training and testing samples. The training dataset serves as the training ground for the CA U-net network, embodying three integral modules—feature extraction, cross-attention module, and road generation module. (3) Accuracy assessment. The roads generated through training samples are then compared with the testing samples derived from original OSM roads for accuracy evaluation. We further use two traditional methods – optimal threshold (OT) method and support vector machines (SVM) method to extract roads from the same training samples, and then compare their extraction accuracy with CA U-net model.

*5. Generalized research method workflow is missing*

**Our response:** Thank you for your helpful comments. We have provided two new figures to introduce the whole road extraction process (Figure 2) and our deep learning framework (Figure 4), respectively. Detailed descriptions can be found in Section 2.2 (Line 195-234) and Section 2.5 (Line 288-304).

**Figure 2**. Flowchart of the road extraction process.

**Figure 4.** The proposed CA U-Net framework for road extraction.

*6. Software and/programs used in the research are not included.*

**Our response:** Thanks for your comments. We have added related content about the details, like software, platforms, computing times, in the *Results* section. Evidence is as bellow:

**Section 3.2 (Line 399-410):** We conducted our analysis predominantly utilizing the ArcGIS Pro 3.0.1 platform, capitalizing on its robust spatial analysis module and accompanying Python library. These tools not only facilitate comprehensive analysis but also allow for secondary development. Within the ArcGIS Pro deep learning framework, we integrated the cross-attention algorithm into the U-Net module layers. This integration was achieved using the secondary development tool, ArcPy. Subsequently, we employed this newly developed module to train a dataset comprising 1,022 labeled image pairs. The training procedure required approximately 28,376 seconds, averaging 27.76 seconds per image pair. Furthermore, the SVM algorithm was implemented via ArcPy within the ArcGIS Pro environment, consuming roughly 245 seconds for the entire categorization process. Lastly, we applied the OT method using the raster calculator feature in ArcGIS Pro, which required a mere 62 seconds upon selecting the appropriate thresholds.

7. The paper lacks a thorough comparison of the cited works and a detailed discussion of the findings.

**Our response:** Thanks for your helpful suggestions. We have made major revisions of the discussion part. We have discussed the comparison with previous NTL data sources and explored the potentiality of combining different approaches for better road extraction accuracy. We have rewritten the whole *Discussion* section according to your comments. And here we just put some key findings in this response letter, more details please refer to the *Discussion* section:

**4.1 Advantages of Yangwang 1 images compared with other NTL platforms**

(Line 422-434) Nighttime light (NTL) data is an emerging remote sensing source extensively employed in socio-economic research[13]. Earlier studies primarily used DMSP/OLS and NPP/VIIRS NTL data, with relatively low resolution[43]. Consequently, these data sources are limited to addressing macro-level issues such as economic development and population dynamics. The procurement of high-resolution NTL data poses significant economic and logistical challenges, resulting in fewer investigations involving high-resolution nighttime light imagery. Yangwang-1 NTL data, characterized by higher resolution, multi-band range, and low cost, can furnish significant advantages for micro-urban research, such as urban traffic evaluation. In order to compare the extraction accuracy of different NTL images, we acquired the Luojia-1 NTL image and 2020 annual NPP/VIIRS image of Wenzhou. Due to the life cycle of Luojia-1 satellite, the newest NTL data we can obtain is collected in October, 2018. The comparison of the extraction accuracy is shown in Figure 8.

**Figure 8**. Accuracy comparison between Yangwang-1 images and VIIRS, Luojia-1 images.

**4.2 Potentiality of combined framework**

Within Wenzhou City, both road extraction methodologies based on Yangwang-1 NTL data yielded F1 scores above 70%, with the CA U-Net model achieving an impressive 84.46%. This highlights its efficacy for road extraction using NTL data. To validate the model's universality, we selected three representative locations in Wenzhou. Comparisons revealed that roads extracted via the CA U-Net framework more accurately corresponded to the reference road network than those extracted by SVM and OT. The SVM model exhibited satisfactory extraction accuracy in suburban areas but struggled in densely lit urban regions compared to CA U-Net. OT performed poorly across all regions, suggesting its unsuitability for standalone road extraction. However, we also realized that OT method can be combined with CA U-Net during the road extraction procedure, because the proper selection of threshold will help with the screen of background noises and blooming effects, which will decrease the accuracy of training samples. In order to test our hypothesis, we overlay the OSM roads with Yangwang-1 NTL data and find that the DN values of all the road pixels are within the range (1, 3325). According to the percentage of road pixels, we chose several ranges and use that to train the CA U-Net model. Their extraction accuracy is shown in Table 2.

**Table 2.** F1 scores of CA U-Net, SVM, and OT models.

| **Lower threshold** | **Upper threshold** | **Percentage of road pixels(%)** | **Precision rate (%)** | **Recall rate (%)** | **F1 Score (%)** |
| --- | --- | --- | --- | --- | --- |
| 1 | 3325 | 100 | 87.51 | 81.62 | 84.46 |
| 168 | 2689 | 95 | 90.64 | 80.54 | 85.29 |
| 426 | 2073 | 90 | 92.15 | 73.85 | 81.99 |
| 535 | 1894 | 85 | 92.65 | 72.76 | 81.51 |

*8. CONCLUSIONS is more of an afterthought. The authors are suggested to include afterthought of this work.*

*9. Recommend some avenues for future research at the end of the conclusion section*

**Our response:** Thanks for your helpful suggestions. We have rewritten the whole *Conclusion* section with better statement of our main findings, more afterthoughts of this study and better suggestions for future work.

**Discussion section (Line 490-527):** Nighttime light (NTL) remote sensing data introduces an innovative perspective to road extraction, traditionally dominated by daytime satellite imagery. This research explores the potentiality of Yangwang-1 NTL data for urban road extraction with the combination of OSM roads through a novel deep learning framework, leading to several significant conclusions.

(1) Yangwang-1 data emerges as a viable source for urban road extraction. As a new generation of NTL data set in ~30 resolution, Yangwang-1 has been proven to have the potentiality to contain detailed road segmentation information through its wide DN range and good data quality. Through the experiment in Wenzhou, we find that Yangwang-1 has higher extraction accuracy in suburban and residential area and may contain a few classification errors in urban center with blooming effects and rural area with limited light intensity.

(2) We propose a novel deep learning framework CA U-Net to train the NTL data with the reference of OSM roads. It achieves an F1 score of 84.46% for road extraction, surpassing SVM and OT performance, thereby underscoring the potential of deep learning frameworks in road extraction tasks using NTL data. Through the application of SVM, OT, and the CA U-Net framework for road extraction in the Wenzhou region based on NTL data, we observe F1 scores exceeding 70% for both SVM and the CA U-Net framework. This also reinforces the suitability of Yangwang-1 data for road extraction tasks.

(3) We compare the Yangwang-1 extraction results with NPP/VIIRS and Luojia-1 images, the results indicate the accuracy of Yangwang-1 images is highest (87.51%). We have also discussed the potentiality of combining OT method with deep leaning framework. The results show that proper chosen of threshold will effectively screen unrelated noises or abnormal pixels, thus improving the extraction accuracy.

This research is a very preliminary exploration of Yangwang-1 NTL images in the road extraction tasks with the expectation to reduce the cost of traditional road extraction through VHR images. Therefore, there are some limitations that need to be further addressed in future studies. First, the blooming effects in city center and feeble light intensity in rural area is a major challenge for the current road extraction through NTL data. One potential solution may be the introduce of moderate-resolution daytime satellite images with low cost, like Landsat images as an information supplementation. That combination may require careful design of approaches to deal with the different data structures of them. Second, the road information is not fully made use of in our deep learning framework. We just use the OSM roads to form the road pixels and non-road pixels in the training process without using its graph information—which contain the connection information between roads. And that information will make our model have the ability to infer road segments which are partly sheltered. Finally, as we have mentioned before, there exist some noises in NTL data and OT approach can be a simple and easy way to preliminarily screen these noises before the conduction of deep learning framework. However, how to chose the best thresholds and combine deep learning technologies with traditional approaches is also a challenge job.

I believe that the modifications made have enhanced the manuscript, making it better suited for publication in PLoS ONE. Enclosed, please find the revised manuscript along with a marked-up copy highlighting the changes made.

Thank you for considering our manuscript for publication in PLoS ONE. I look forward to hearing from you soon.

Sincerely,

Anfeng Zhu,

College of Artificial Intelligence, Zhejiang College of Security Technology,

20096344@zjcst.edu.cn
